# Transcranial Sonography in the Examination of Atypical Parkinsonian Syndromes

**DOI:** 10.3390/biomedicines14030530

**Published:** 2026-02-27

**Authors:** Piotr Alster, Bartosz Migda, Michał Kutyłowski, Michał Markiewicz, Natalia Madetko-Alster

**Affiliations:** 1 Department of Neurology, Medical University of Warsaw, 03-242 Warsaw, Poland; micmar@onet.eu (M.M.); natalia.madetko@wum.edu.pl (N.M.-A.); 2 Diagnostic Ultrasound Lab, Department of Pediatric Radiology, Medical University of Warsaw, 03-242 Warsaw, Poland; bartosz.migda@wum.edu.pl; 3 Department of Radiology, Mazovian Brodno Hospital, 03-242 Warsaw, Poland; michael.kutylowski@gmail.com

**Keywords:** TCS, sonography, atypical parkinsonisms, neuroimaging

## Abstract

Transcranial sonography is one of the methods of examination used in atypical parkinsonian syndromes. The assessment is not indicated in the diagnostic criteria of entities in this group e.g., Progressive Supranuclear Palsy, Corticobasal Degeneration, Multiple System Atrophy and Dementia with Lewy Bodies. Atypical parkinsonisms are a group of diseases affected by diverse pathologies including alpha-synuclein or tau among others. Recently broader attention was brought to less common atypical parkinsonisms as Perry syndrome. Atypical parkinsonisms are related to poor response to levodopa treatment, rapid deterioration and unfavorable prognosis. Additionally, the entities often overlap in terms of clinical manifestation, especially in the early stages. Though atypical parkinsonisms are affected by the lack of possibility of obtaining definite in vivo diagnosis, growing interest is associated to supplementary evaluations including neuroimaging. Among these methods could be mentioned magnetic resonance imaging, positron emission tomography, single photon emission computed tomography and transcranial sonography. Transcranial sonography is associated with high accessibility and low cost. The goal of this paper is to highlight the strengths and weaknesses of transcranial sonography in the examination of atypical parkinsonisms.

## 1. Introduction

Neuroimaging is a crucial element in the supplementary assessment of parkinsonian syndromes, especially in the differentiation of Parkinson’s diseases (PD) and other causes of resembling clinical manifestations. Though the specificity of neuroimaging gradually increases, the crucial point related to the examination of parkinsonisms relies on clinical. neurological evaluation. Nevertheless global population aging, the human and social costs lead to the necessity for obtaining more efficient tools in the assessment of atypical parkinsonisms and many other neurodegenerative diseases. Parkinsonian syndrome may be caused by multiple factors including genetic, environmental, infectious among others [1]. The most significant obstacle in the differential diagnosis of PD are associated to the misdiagnosis of atypical parkinsonisms. This group comprises of diseases based on multiple pathologies among which could be mentioned alpha-synuclein, tau and transactive response deoxyribonucleic acid binding protein 43 (TDP-43). The entities are linked to ambiguous correlations of clinical symptoms, rapidly evolving deterioration, poor prognosis and the lack of effective treatment. Among methods used in the examination, the most relevant is linked to magnetic resonance imaging (MRI). MRI parameters are considered as possibly feasible in the differentiation of PD an atypical parkinsonisms. In this group could be mentioned the hummingbird sign linked to mesencephalic atrophy in Progressive Supranuclear Palsy (PSP), putaminal rim and hot cross bun sign in Multiple System Atrophy (MSA) or asymmetric atrophic changes in Corticobasal Degeneration (CBD) [2]. In case of MRI certain parameters as the evaluation of mesencephalon and pons ratio, magnetic resonance parkinsonism index (MRPI) and its evolved version (MRPI 2.0) revealed usefulness in the examination of atypical parkinsonisms [3]. Growing interest is associated to the significance of single photon emission computed tomography (SPECT) and positron emission tomography (PET) with the use of tau-radiotracers [4]. In this group could be mentioned first generation radiotracers as 18-F-AV1451 [5]. This group showed encouraging results in the examination of Alzheimer’s disease, however in the evaluation of atypical parkinsonisms with tau pathology, the radiotracers are affected by multiple off-binding properties among which could be mentioned the affinity to monoaminoxidase, hemorrhagic focuses and neuromelanin. Growing interest is associated to second generation radiotracers as 18-F-PI2620 which are linked to higher specificity, enabling indication of neuronal and oligodendroglial tau [6]. Methods linked to nuclear medicine as PET and SPECT, though enabling indication of pathological proteins or abnormalities of perfusion and metabolism often preceding the initiation of symptoms, are affected by off binding properties, moreover their accessibility due to relatively high costs is limited. Another method, highly accessible, is linked to transcranial sonography. Its’ possible utility is widely indicated in the context of PD, less attention is brought up in terms of atypical parkinsonisms, a diverse group of diseases comprising PSP, MSA, CBD and Dementia with Lewy Bodies (DLB). Though the contemporary criteria of diagnosis provide increasingly precise description of the manifestations, the diseases are generally linked to the lack of possibility of obtaining in vivo definite diagnosis. Due to the overlaps in clinical manifestation of the diseases, effective supplementary examinations are necessary. Certain clinical feature may be present in multiple entities in the group of atypical parkinsonisms [7,8,9,10]. Additionally the possible pathogenesis of entities resembling atypical parkinsonisms apart from neurodegeneration, may be related to vascular, infectious, environmental factors among others [1]. In this context transcranial sonography may seem as a neuroimaging method related to low cost and high accessibility. Growing interest is related to the specificity and sensitivity of the method, which is not mentioned in the contemporary criteria of diagnosis of PSP, CBD, MSA and DLB [7,8,9,10]. The aim of this article is to elaborate on strengths and weaknesses associated with transcranial sonography, a screening and non-invasive tool, in the evaluation of parkinsonisms including atypical parkinsonian syndromes [11,12].

## 2. Analysis of TCS Significance in Atypical Parkinsonisms

### 2.1. Dementia with Lewy Bodies

DLB, one of the three most common causes of dementia, is associated with bilateral marked hyperechogenicity of the substantia nigra, which is considered as a differential factor of Alzheimer’s disease (AD) [13]. The evaluation of this parameter is considered as feasible in the early evaluation of dementias, however no correlation with Unified Parkinson’s Disease Rating Scale part III, Mini Mental State Examination or clinical features of DLB as the presence of visual hallucinations were found [14]. In the differential diagnosis of DLB with AD and PD, the hyperechogenicity within the substantia nigra accompanied by the widening of the frontal horns of lateral ventricles is considered as a predictive factor in diagnosing early stage DLB [15]. Increased echogenicity in the substantia nigra observed bilaterally is considered as valuable parameter in the sonographic evaluation of dementias as a relevant factor in DLB [16]. A sonographic evaluation of DLB, Parkinson’s disease with dementia (PDD) and PD without dementia revealed abnormalities. Increased width of third ventricle and frontal horns showed differential potential between PD with PDD and DLB [17]. The most efficient differentiation in terms of sensitivity between PDD and DLB was obtained with the combined assessment of echogenic sizes, asymmetry indices and onset age.

### 2.2. Progressive Supranuclear Palsy

In PSP the examination using transcranial sonography is affected by diverse results depending on the subtype of the disease. This particular feature becomes an obstacle in the evaluation of the second major subtype of the disease—PSP-Parkinsonism Predominant (PSP-P) in which supplementary examinations enabling differentiation with PD seem crucial. In a study based on the examination of 27 patients with PSP-Richardson’s syndrome (PSP-RS) and 7 patients with PSP-P, hyperechogenic substantia nigra was detected in most cases in PSP-P—86%, whereas in the case of PSP-RS the vast majority presented normal echogenicity in this structure [18]. A different assessment based on the evaluation of 11 patients with PSP-P and 21 with PSP-RS showed higher incidence of hyperechogenic substantia nigra in PSP-P (73%), when compared to PSP-RS (14%) [19]. The same study showed hyperechogenicity of the lentiform nucleus as a more common feature of PSP-RS when compared to PSP-P. The width of the third ventricle was found to be significantly higher in PSP-RS than in PSP-P. The observation concerning the third ventricle comes up with examination using other methods of neuroimaging [20]. A meta-analysis based on the assessment of 366 patients showed common association of PSP with lenticular nucleus hyperechogenicity and the increase of the width of the third ventricle [21]. A different study highlighting the transcranial evaluations of mesencephalic area in the differentiation of parkinsonisms revealed significantly decreased values in PSP when compared to PD [22]. In this analysis authors combined this examination with the assessment of the width of the third ventricle, which was significantly higher in PSP. Interestingly, as the study confirmed wider third ventricles in PSP-RS than in PSP-P, the areas of the mesencephalon evaluated using this method, did not show significant discrepancies. Evaluations of PD and PSP patients using sonographic measurements of the mesencephalon indicated the reduced in PSP. Although the evaluations of the larger area of mesencephalon was a more sensitive indicator of PD, in the context of PSP examination, the increased width of the third ventricle was interpreted as a more sensitive parameter [23]. Other evaluations of PSP using transcranial sonography as the verification of hyperechogenic lentiform nucleus, provide efficient tools in the differentiation of PD and atypical parkinsonisms, however their differential potential between PSP with other atypical parkinsonisms as MSA is ambiguous [24,25,26]. The hyperechogenic lentiform nucleus and increased width of the third ventricle are a repeatedly brought up feature in PSP also in other studies [27].

### 2.3. Multiple System Atrophy

In MSA the assessment of substantia nigra using transcranial sonography shows limited significance in the differentiation with non-tremor dominant PD. A study based on the examination of 86 MSA-Parkinsonism Dominant and 147 age and gender-matched PD patients revealed limited specificity and sensitivity in the differential diagnosis of early-stage diseases [28]. The lack of significant abnormalities regarding the echogenicity of substantia nigra in MSA is a repeatedly highlighted feature [29]. A relatively large Chinese study based on the examination of 375 patient with PD, MSA, PSP and ET, showed lower echogenicity within the substantia nigra in MSA when compared to PD, however this observation did not significantly differ atypical parkinsonisms [25]. Comparisons of the lenticular nucleus showed lower echogenicity in MSA than in PD and PSP [25]. Normal echogenicity of the substantia nigra combined with hyperechogenic lenticular nucleus is considered as possible indication of MSA or PSP. Moreover, the combined presence of parkinsonism with the onset before the age of 60 and lack of abnormalities in the context of substantia nigra echogenicity is mentioned as a possible factor in favor of the two mentioned diseases [30].

### 2.4. Corticobasal Degeneration

CBD and Corticobasal Syndrome (CBS) are entities which are less verified in terms of transcranial sonography due to obstacles in clinical evaluation. In a study based on eight cases of CBD, the disease was linked to hyperechogenicity in the substantia nigra [31]. Hyperechogenic substantia nigra is considered as a supportive feature in differentiating CBD with PSP [32]. Among other features of CBD, authors of one of the research projects mentioned the width of the third ventricle not exceeding 10 mm. This threshold was associated to a sensitivity of 100%, specificity of 83% and positive value of 80% in the differentiation with PSP [31]. CBS due to its diverse pathology, which may be associated to PSP, Frontotemporal Dementia or Alzheimer’s disease, is a clinical syndrome with ambiguous background.

### 2.5. Other Atypical Parkinsonisms

Transcranial sonography was also performed in very rare, atypical parkinsonisms as Perry syndrome associated with rapidly progressing parkinsonian syndrome, depression, apathy, dyspnea and weight loss. The syndrome is based on TDP-43 pathology [33]. In a study evaluating two siblings, this examination revealed hyperechogenicity in the substantia nigra [33]. The observation regarding substantia nigra was confirmed in other studies [34]. The abnormalities are considered as nigral degeneration sign not necessarily linked to particular pathology. The TCS presentation of substantia nigra shows resemblance to PD.

## 3. Transcranial Sonography in Combined Evaluations

In the examination of atypical parkinsonisms growing interest is associated with the combination of sonographic evaluations. In a multicenter study assessing atypical parkinsonisms authors analyzed the possible relevance of parallel examinations of substantia nigra hyperechogenicity, lenticular nucleus hyperechogenicity and third ventricle enlargement [35]. The evaluation of at least two parameters showed higher specificity and lower sensitivity in PD as well as atypical parkinsonisms. A different study based on the evaluation of combined transcranial sonography and positron emission tomography and 18-fluorodeoxyglucose showed similar levels of accuracy in the evaluation of parkinsonisms. The sonographic assessment was based on the analyzes of hyperechogenicity within the substantia nigra and lenticular nucleus and the width of the third ventricle. The study examined a relatively low number of patients with atypical parkinsonism–21. Moreover, each of the groups within the atypical parkinsonisms comprised of 5–9 patients with the diagnoses of PSP, MSA and CBS, which significantly downscores the outcome of differentiation between those entities [36]. Combined evaluation of echogenicity within the substantia nigra and lentiform nucleus was a factor indicated in an evaluation of 102 patients with PD, 34 with MSA and 21 with PSP. The dual evaluation showing hyperechogenic substantia nigra and regular echogenicity in the lentiform nucleus was suggestive of PD and showed positive predictive value of 0.91. Hyperechogenic lentiform nucleus accompanied by moderate echogenic substantia nigra was suggestive of atypical parkinsonian syndromes and showed positive predictive value of 0.96. The positive predictive values in this research were associated with the examinations of 88 patients with PD and 50 with atypical parkinsonisms. The limited number of examined patients was related to the rarity of PSP and MSA and partly the lack of sufficient temporal bone window. In this work by Behnke et al a percentage ranging between 25 and 39% was indicated in the context of marked substantia nigra in atypical parkinsonian syndromes, which based on the outcomes of other studies, may be a consequence of the lack of subanalysis of subtypes of PSP as PSP-RS and PSP-P may significantly differ [37]. The methodological limitations of the study highlighted possible tools of supplementary examinations rather than definitive indicators.

## 4. Limitations

Transcranial sonography is affected by certain limitations. The main one is associated to the lack of appropriate temporal acoustic bone windows. Certain obstacles may be related with the deviations in terms of cooperation with the patient. Thirdly, the limited specificity of the method may downscore the significance of repeatable assessment. The comparability between the evaluations using transcranial sonography may be affected by the physician performing the examination, moreover the assessment of evolution of certain abnormalities is bounded by the accuracy. Certain studies are performed as a differentiation between PD and non-PD patients. In this context studies in which non-PD group comprise of atypical parkinsonisms and essential tremor, downscore the possible differential analysis [12]. Recent studies additionally highlight that especially in the context of PSP, joined evaluation of subtypes may jeopardize the outcome of evaluations. Moreover, certain coexisting factors as depression may influence the hyperechogenicity in the atypical parkinsonisms [38]. Depression should be interpreted as an example, however the presence of comorbidities may impact the outcome of transcranial sonography evaluation. 

## 5. Conclusions

Transcranial sonography shows promising results in the combined evaluations of multiple parameters assessed using this method (Table 1). It should be considered as a supplementary tool interpreted with increased caution in the case of atypical parkinsonisms, as the subtypes may differ, moreover overlaps in clinical manifestation cause significant obstacles in the interpretation of the results of transcranial sonography.

## Figures and Tables

**Table 1:** Transcranial evaluation of atypical parkinsonisms.

	DLB	PSP	MSA	CBD	Perry Syndrome
Substantia nigra	(+)	(−) in PSP-RS (+) in PSP-P	(−)	(+)	(+)
Lentiform nucleus	(−)	(+)	(+)	(+)	No sufficient data
Width of the third ventricle	(+)	(+)	(−)	(−)	No sufficient data

Abbreviations: CBD—Corticobasal Degeneration, DLB—Dementia with Lewy Bodies, MSA—Multiple System Atrophy, PSP—Progressive Supranuclear Palsy. (+) increased, (−) without significant abnormalities.

## Data Availability

No new data were created or analyzed in this study. Data sharing is not applicable to this article.

## References

[1] Koziorowski D., Figura M., Milanowski Ł.M., Szlufik S., Alster P., Madetko N., Friedman A. (2021). Mechanisms of Neurodegeneration in Various Forms of Parkinsonism-Similarities and Differences. Cells.

[2] Saeed U., Lang A.E., Masellis M. (2020). Neuroimaging Advances in Parkinson’s Disease and Atypical Parkinsonian Syndromes. Front. Neurol..

[3] Quattrone A., Bianco M.G., Antonini A., Vaillancourt D.E., Seppi K., Ceravolo R., Strafella A.P., Tedeschi G., Tessitore A., Cilia R. (2022). Development and Validation of Automated Magnetic Resonance Parkinsonism Index 2.0 to Distinguish Progressive Supranuclear Palsy-Parkinsonism From Parkinson’s Disease. Mov. Disord..

[4] Alster P., van Eimeren T., Madetko-Alster N. (2025). Tau positron emission tomography in the Parkinson and Richardson subtypes of progressive supranuclear palsy. Park. Relat. Disord..

[5] Alster P., Madetko N.K., Koziorowski D.M., Królicki L., Budrewicz S., Friedman A. (2019). Accumulation of Tau Protein, Metabolism and Perfusion-Application and Efficacy of Positron Emission Tomography (PET) and Single Photon Emission Computed Tomography (SPECT) Imaging in the Examination of Progressive Supranuclear Palsy (PSP) and Corticobasal Syndrome (CBS). Front. Neurol..

[6] Slemann L., Gnörich J., Hummel S., Bartos L.M., Klaus C., Kling A., Kusche-Palenga J., Kunte S.T., Kunze L.H., Englert A.L. (2024). Neuronal and oligodendroglial, but not astroglial, tau translates to in vivo tau PET signals in individuals with primary tauopathies. Acta Neuropathol..

[7] Höglinger G.U., Respondek G., Stamelou M., Kurz C., Josephs K.A., Lang A.E., Mollenhauer B., Müller U., Nilsson C., Whitwell J.L. (2017). Clinical diagnosis of progressive supranuclear palsy: The movement disorder society criteria. Mov. Disord..

[8] Armstrong M.J., Litvan I., Lang A.E., Bak T.H., Bhatia K.P., Borroni B., Boxer A.L., Dickson D.W., Grossman M., Hallett M. (2013). Criteria for the diagnosis of corticobasal degeneration. Neurology.

[9] Wenning G.K., Stankovic I., Vignatelli L., Fanciulli A., Calandra-Buonaura G., Seppi K., Palma J., Meissner W.G., Krismer F., Berg D. (2022). The Movement Disorder Society Criteria for the Diagnosis of Multiple System Atrophy. Mov. Disord..

[10] McKeith I.G., Boeve B.F., Dickson D.W., Halliday G., Taylor J.-P., Weintraub D., Aarsland D., Galvin J., Attems J., Ballard C.G. (2017). Diagnosis and management of dementia with Lewy bodies: Fourth consensus report of the DLB Consortium. Neurology.

[11] Vlaar A.M., de Nijs T., van Kroonenburgh M.J., Mess W.H., Winogrodzka A., Tromp S.C., Weber W.E. (2008). The predictive value of transcranial duplex sonography for the clinical diagnosis in undiagnosed parkinsonian syndromes: Comparison with SPECT scans. BMC Neurol..

[12] Stiehm M., Nilsson C., Skogar Ö., Walter U. (2025). The diagnostic value of transcranial sonography in Swedish parkinsonism patients: A retrospective cohort study with long-term follow-up. Clin. Park. Relat. Disord..

[13] Favaretto S., Walter U., Baracchini C., Cagnin A. (2018). Transcranial Sonography in Neurodegenerative Diseases with Cognitive Decline. J. Alzheimer’s Dis..

[14] Favaretto S., Walter U., Baracchini C., Pompanin S., Bussè C., Zorzi G., Ermani M., Cagnin A. (2016). Accuracy of transcranial brain parenchyma sonography in the diagnosis of dementia with Lewy bodies. Eur. J. Neurol..

[15] Planas-Ballvé A., Rios J., Gea M., Rabaneda-Lombarte N., Ispierto L., Grau L., Jiménez M., Cáceres C., Martínez S., Beyer K. (2024). Substantia nigra hyperechogenicity and brain ventricular size as biomarkers of early dementia with Lewy bodies. Alzheimer’s Res. Ther..

[16] Santos R.P., Fernandes R.d.C.L. (2025). State-of-the-Art Transcranial Sonography for Neuropsychiatric and Dementia Disorders: A Narrative Review. Cureus.

[17] Walter U., Dressler D., Wolters A., Wittstock M., Greim B., Benecke R. (2006). Sonographic discrimination of dementia with Lewy bodies and Parkinson’s disease with dementia. J. Neurol..

[18] Ebentheuer J., Canelo M., Trautmann E., Trenkwalder C. (2010). Substantia nigra echogenicity in progressive supranuclear palsy. Mov. Disord..

[19] Kostić V.S., Mijajlović M., Smajlović D., Lukić M.J., Tomić A., Svetel M. (2013). Transcranial brain sonography findings in two main variants of progressive supranuclear palsy. Eur. J. Neurol..

[20] Wen Y., Yang Q., Jiao B., Zhang W., Lin J., Zhu Y., Xu Q., Zhou H., Weng L., Liao X. (2023). Clinical features of progressive supranuclear palsy. Front. Aging Neurosci..

[21] Wen Y., Zhou H., Xia M., Liu Q., Quan H., Fang L. (2024). Differentiating progressive supranuclear palsy from other movement disorders using transcranial sonography: A systematic review and meta-analysis. Neurol. Sci..

[22] Sastre-Bataller I., Vázquez J.F., Martínez-Torres I., Sahuquillo P., Rubio-Agustí I., Burguera J.A., Ferrer J.M., Valero C., Tembl J.I. (2013). Mesencephalic area measured by transcranial sonography in the differential diagnosis of parkinsonism. Park. Relat. Disord..

[23] Ghourchian S., Mousavi A., Zamani B., Shahidi G., Rohani M. (2019). Midbrain area for differentiating Parkinson’s disease from progressive supranuclear palsy. Clin. Neurol. Neurosurg..

[24] Richter D., Katsanos A.H., Schroeder C., Tsivgoulis G., Paraskevas G.P., Müller T., Alexandrov A.V., Gold R., Tönges L., Krogias C. (2019). Lentiform Nucleus Hyperechogenicity in Parkinsonian Syndromes: A Systematic Review and Meta-Analysis with Consideration of Molecular Pathology. Cells.

[25] Wang L.-S., Yu T.-F., Chai B., He W. (2021). Transcranial sonography in differential diagnosis of Parkinson disease and other movement disorders. Chin. Med. J..

[26] Bouwmans A.E., Vlaar A.M., Srulijes K., Mess W.H., Weber W.E. (2010). Transcranial sonography for the discrimination of idiopathic Parkinson’s disease from the atypical parkinsonian syndromes. Int. Rev. Neurobiol..

[27] Sanzaro E., Iemolo F. (2016). Transcranial sonography in movement disorders: An interesting tool for diagnostic perspectives. Neurol. Sci..

[28] Zhou H.-Y., Huang P., Sun Q., Du J.-J., Cui S.-S., Hu Y.-Y., Zhan W.-W., Wang Y., Xiao Q., Liu J. (2018). The role of substantia nigra sonography in the differentiation of Parkinson’s disease and multiple system atrophy. Transl. Neurodegener..

[29] Smajlovic D., Ibrahimagic O.C. (2017). Transcranial Brain Sonography in Parkinson’s Disease and Other Parkinsonian Disorders: A Hospital Study from Tuzla, Bosnia and Herzegovina. Med. Arch..

[30] Walter U., Dressler D., Probst T., Wolters A., Abu-Mugheisib M., Wittstock M., Benecke R. (2007). Transcranial brain sonography findings in discriminating between parkinsonism and idiopathic Parkinson disease. Arch. Neurol..

[31] Walter U., Dressler D., Wolters A., Probst T., Grossmann A., Benecke R. (2004). Sonographic discrimination of corticobasal degeneration vs progressive supranuclear palsy. Neurology.

[32] Gaenslen A., Unmuth B., Godau J., Liepelt I., Di Santo A., Schweitzer K.J., Gasser T., Machulla H.-J., Reimold M., Marek K. (2008). The specificity and sensitivity of transcranial ultrasound in the differential diagnosis of Parkinson’s disease: A prospective blinded study. Lancet Neurol..

[33] Saka E., Topcuoglu M.A., Demir A.U., Elibol B. (2010). Transcranial sonography in Perry syndrome. Park. Relat. Disord..

[34] Mishima T., Fujioka S., Tomiyama H., Yabe I., Kurisaki R., Fujii N., Neshige R., A Ross O., Farrer M.J., Dickson D.W. (2018). Establishing diagnostic criteria for Perry syndrome. J. Neurol. Neurosurg. Psychiatry.

[35] Alonso-Canovas A., Ferrairó J.I.T., Martínez-Torres I., Moreno J.L.L.-S., Parees-Moreno I., Monreal-Laguillo E., Pérez-Torre P., Delgado R.T., Ribas G.G., Bataller I.S. (2019). Transcranial sonography in atypical parkinsonism: How reliable is it in real clinical practice? A multicentre comprehensive study. Park. Relat. Disord..

[36] Hellwig S., Reinhard M., Amtage F., Guschlbauer B., Buchert R., Tüscher O., Weiller C., Niesen W.D., Meyer P.T. (2014). Transcranial sonography and [^18^F]fluorodeoxyglucose positron emission tomography for the differential diagnosis of parkinsonism: A head-to-head comparison. Eur. J. Neurol..

[37] Behnke S., Berg D., Naumann M., Becker G. (2005). Differentiation of Parkinson’s disease and atypical parkinsonian syndromes by transcranial ultrasound. J. Neurol. Neurosurg. Psychiatry.

[38] Bouwmans A.E., Weber W.E., Leentjens A.F., Mess W.H. (2016). Transcranial sonography findings related to depression in parkinsonian disorders: Cross-sectional study in 126 patients. PeerJ.

